# Antimicrobial Stewardship Activities in Public Healthcare Facilities in South Africa: A Baseline for Future Direction

**DOI:** 10.3390/antibiotics10080996

**Published:** 2021-08-17

**Authors:** Deirdré Engler, Johanna Catharina Meyer, Natalie Schellack, Amanj Kurdi, Brian Godman

**Affiliations:** 1 Division of Public Health Pharmacy and Management, School of Pharmacy, Sefako Makgatho Health Sciences University, Ga-Rankuwa 0204, South Africa; hannelie.meyer@smu.ac.za (J.C.M.); Amanj.Baker@strath.ac.uk (A.K.); Brian.Godman@strath.ac.uk (B.G.); 2 Department of Pharmacology, University of Pretoria, Arcadia 0007, South Africa; natalie.schellack@up.ac.za; 3 Strathclyde Institute of Pharmacy and Biomedical Sciences (SIPBS), Strathclyde University, Glasgow G4 0RE, UK; 4 Department of Pharmacology, College of Pharmacy, Hawler Medical University, Erbil, Iraq; 5 School of Pharmaceutical Sciences, University Sains Malaysia, George Town 118000, PNG, Malaysia

**Keywords:** antimicrobial resistance, antimicrobial stewardship programmes, primary healthcare, South Africa

## Abstract

Antimicrobial resistance (AMR) is a growing problem worldwide, including South Africa, where an AMR National Strategy Framework was implemented to instigate antimicrobial stewardship programmes (ASPs) and improve antimicrobial prescribing across sectors. To address the need to assess progress, a sequential mixed methodology with an explanatory research design was employed. In Phase 1, a self-administered questionnaire was completed by healthcare professionals (HCPs) from 26 public sector healthcare facilities across South Africa to assess compliance with the Framework. The results were explored in Phase 2 through 10 focus group discussions and two in-depth interviews, including 83 participants. Emerging themes indicated that public healthcare facilities across South Africa are facing many challenges, especially at entry level primary healthcare (PHC) facilities, where antimicrobial stewardship activities and ASPs are not yet fully implemented. Improved diagnostics and surveillance data are a major shortcoming at these facilities. Continuous education for HCPs is deficient, especially for the majority of prescribers at PHC level and health campaigns are nearly non-existent. Involvement and visibility of management at certain facilities is a serious shortfall. Consequently, it is important to call attention to the challenges faced with improving antimicrobial prescribing across countries and address these to reduce AMR, especially in PHC facilities, being the first point of access to healthcare for the vast majority of patients in developing countries.

## 1. Introduction

The globe is facing an immense challenge with the spread of resistant antimicrobial pathogens [1], with antimicrobial resistance (AMR) increasing morbidity, mortality and costs [2,3,4,5]. Infections account for the major burden of disease among African countries with high rates of community and hospital-acquired infections, including HIV and TB [6]. Extensive use of antimicrobials inevitably leads to AMR [7,8,9,10], with AMR now present in every country including African countries and considered a global public health crisis [11,12]. Whilst we are aware that improvement in healthcare through increased resources is becoming a priority across countries, including many low- and middle-income countries (LMICs), there is still considerable concern about lack of investment in managing both infectious and non-infectious diseases although this is changing [13,14,15,16,17,18,19].

An area of critical importance is to safeguard the use of antimicrobials and to focus on appropriate and targeted antimicrobial use in humans, animals and the environment [20,21]. This is the strategy behind the World Health Organizations’ One Health approach [22,23]. We have seen investment in multiple strategies improve antibiotic utilization among LMICs providing direction to others [10,24,25,26]. This includes Kazakhstan where multiple strategies helped reduce inpatient antibiotic utilization from 12.72 DIDs (DDDs/1000 inhabitants/day) in 2011 to 2.74 in 2018 [10] and Slovenia where multiple strategies reduced overall antibiotic utilization by 31% between 1999 and 2012 [24]. However, in Poland, the lack of multiple coordinated activities resulted in a small but statistically significant annual increase in total antibiotic consumption from 22.2 DIDs to 23.9 DIDs between 2007 and 2016 [21,27].

South Africa pledged its commitment to the World Health Assembly resolution EB134/37 ‘Combating AMR including antibiotic resistance’, adopted in May 2014, to develop a National Action Plan on AMR [28]. This resulted in the June 2017 implementation plan for the Antimicrobial Resistance National Strategy Framework in South Africa 2014–2024. The country is also currently in the process of implementing a National Health Insurance (NHI) system in its move towards universal health coverage (UHC) to improve access and affordability of healthcare to all [15,19,29].

According to the 2018 General Household Survey, as many as 47 million South Africans do not have the privilege of private medical insurance [30]. Consequently, the majority of the population in South Africa use government funded local clinics, community health centres (CHCs) or hospitals as entry level to the healthcare system, when ill or injured, and in need of medical assistance [30,31,32]. From this primary healthcare (PHC) level, which includes local clinics and CHCs [33], patients can only access higher levels of care (secondary and tertiary) upon assessment and referral by health care professionals (HCPs) from a lower level [29,31,33].

Developing countries, also referred to as LMICs, are implicated in the inappropriate prescribing of medicines in healthcare settings including antimicrobials for essential viral infections [21,34,35]. The current dilemma regarding AMR is due to the overuse and inappropriate use of antimicrobials, which includes inappropriate selection and dosing by HCPs, and non-adherence to treatment guidelines including among HCPs in South Africa [36,37,38,39,40,41,42]. The WHO in collaboration with the International Network for Rational Use of Drugs recommend proper documentation of medicine use, applying core indicators (34). However, there are concerns whether these actually measure the quality of antimicrobial prescribing in ambulatory care, with reference to better clinical outcomes [21,36].

Currently, there is a paucity of scientific data pertaining to the African region on the quality of antimicrobial use at the PHC level, including adherence to prescribing guidelines which is considered a good indicator of the quality of prescribing [21,38,39,42,43]. Consolidating programmes and initiatives including antimicrobial stewardship (AMS) across health sectors is necessary to address AMR [44,45,46]. However, it is recognized that instigating antimicrobial stewardship programmes (ASPs) are more challenging in LMICs including sub-Saharan Africa due to manpower and resource issues, as well as knowledge regarding the terminology [47,48,49,50]. The latter is however starting to be addressed through initiatives in South Africa and wider [21,51,52,53,54,55,56].

Consequently, in view of the urgency to reduce AMR world-wide, we believe it is important to describe actions that have been taken among public sector facilities in LMICs especially sub-Saharan African countries to improve future antibiotic use. This includes ambulatory care where the majority of antibiotics are prescribed and dispensed in LMICs [21,35]. In South Africa, this includes activities at PHC level in line with the recommendations in South Africa’s AMR National Strategy Framework, and to explore factors affecting the implementation, success and sustainability of the national strategy. We believe such activities will be of interest to key stakeholder groups not only in South Africa but also wider as LMICs move to the next stage of monitoring progress towards their national action plans (NAPs) to reduce AMR given concerns with the implementation of NAPs among a number of LMICs [21,57,58,59,60,61].

## 2. Results

Of the 37 HCPs invited to participate in Phase 1, 26 (70.3%) agreed and completed the questionnaire [33]. Table 1 shows the respondents distributed by profession and facility type. Participation from the different types of healthcare facilities was equally distributed between central hospitals, referral hospitals and CHCs, with most respondents being pharmacists. Fifteen (57.7%) participants were in managerial positions at their facilities.

A total number of 83 HCPs participated in Phase 2. Two individual in-depth interviews were conducted and three microbiologists that could not attend the discussions, replied to questions sent via e-mail. Table 2 shows participant representation from various disciplines, and the distribution of participants according to duration in a current position.

**Figure 1:**
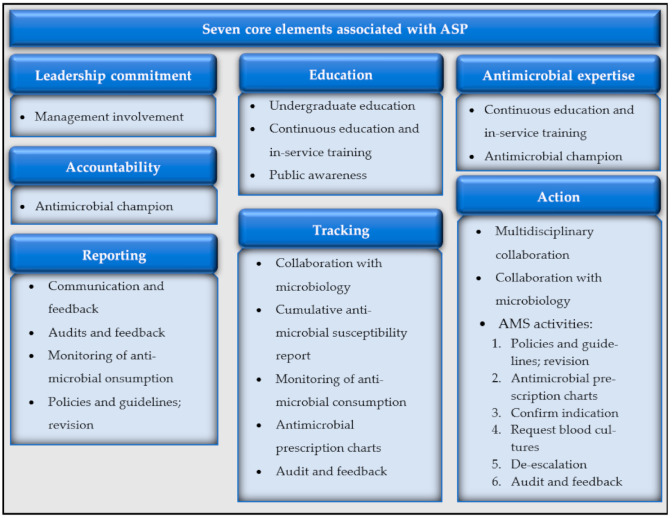
Emerging aspects pertaining to the core elements of ASPs.

Four main themes emerged from the data. For the purpose of this paper, the focus will be on the theme of AMS and ASPs. Figure 1 provides an overview of the seven core elements associated with a successful ASP [21,62,63,64,65], with the themes and sub-themes that emerged from this study.

Although intertwined, the results will be in line with the elements laid out in Figure 1. The results however start off regarding ignorance of the AMR National Strategy Framework and unawareness of AMR at the facility level as this can impact successful AMS and ASPs.

Verbatim quotations from participants to support the findings are presented in italics and are enclosed in inverted commas. Where necessary, essential editing pertaining to grammar and punctuation were used for ease of reading and understanding. The unique identifiers of the various participants are presented in round brackets as ‘P#’ and that of the facilities as ‘F’. Any additional explanations included in the quotations are presented as normal text and enclosed in square brackets.

### 2.1. Awareness of Antimicrobial Resistance

During Phase 1, 19.2% (*n* = 5) of the facilities were of the meaning that AMR does not pose a problem at their facility. This perception was further explored in Phase 2 and it transpired that because facilities do not have the hard facts, they are unaware of AMR. P#30 (pharmacist) from a 24-h CHC (F3) mentioned: “*I think it’s difficult to say because we don’t have evidence if it is yes or no. We don’t have resistance patterns that you can look at … so I don’t think from our side, we can’t say yes or no, because we don’t know*”. Some comments reflected a lack of knowledge and understanding, especially when saying “*Antibiotic resistance is a much greater problem in the first world than in our* [South African] *setting*” (P#26; specialist; F15; district hospital). A critical care specialist’s opinion was that “*People don’t understand the seriousness of the crisis that we face in terms of resistance. They think, aggh, it’s nonsense, and they just sign all these things and they refuse to become involved in the process* [referring to AMS and ASPs].” (P#1; F7; national central hospital).

### 2.2. Awareness of the Antimicrobial Resistance National Strategy Framework

Phase 1 data showed 88.5% (*n* = 23) of HCPs were aware of the Framework, although the average compliance was only 59.5%, ranging from 12.9% to 90.9% (*n* = 26) [33]. One of the district hospitals (F15) used the Framework to establish their AMS committee and was of the opinion that “*it’s a good Framework and it seems like it’s functioning well; our AMS committee is functioning well. And we see progression over the one or two years that I’ve been involved in it*” (P#24; pharmacist). This facility had an 88.2% compliance with the Framework [33]. The flipside of the coin is that this is not necessarily the situation at all public health care facilities, and especially challenging at the PHC level. Even though they might be aware of the Framework, it has not been typically implemented as such. P#29 admitted to have read through the guidelines, “*but our setup at this facility isn’t implementing it according to that Framework yet. It does set out who should be in the team and what procedures have to be followed in order to have the surveillance in place. But yes, we are not doing that at the moment*” (Pharmacy manager; F3; 24-h CHC). The pharmacy manager at another 24-h CHC was of the opinion that “*…you need the tools to implement it—that is the thing*” (P#5; F26).

### 2.3. Education, Communication and Public Awareness as the Basis of the Framework

The foundation of the Framework speaks of ‘education, communication and public awareness’, which all form part of AMS and ASPs. One of the specialists at a national central hospital was of the opinion that public education should be reinforced by legislation: “*…if one looks at health advocacy, public education etc., if I would now just go to for example something like smoking cessation, the only time smoking* i.e., *the number of people smoking reduced was because the legislation was implemented. It wasn’t because you stick a note on a cigarette pack saying smoking kills you, or whatever, vaccination is safe. You leave it to the public to decide on whether it’s done or not. And so with this, it can either be positively reinforced or negatively reinforced in terms of your habits*” (P#11; F6).

#### 2.3.1. Undergraduate Education

Valuable comments were made by various participants regarding undergraduate (UG) training. Many participants could not recall receiving training on antimicrobials, let alone AMR, e.g., the assistant manager of a 24-h CHC said “*I can’t remember that we received something like that* [referring to antimicrobial training and AMR]” (P#31; F3). Another comment by a critical care specialist at a national central hospital, “*They* [referring to doctors] *have no idea* [about antimicrobials]. *I think they* [referring to medical students] *get a week on antibiotics in medical school?*” (P#1; F7), reiterated the lack of training at UG level.

#### 2.3.2. Continuous Education and In-Service Training

During Phase 1 it became evident that only 42% (*n* = 26) of HCPs received continuous education on local AMR. During the focus group discussions (FGDs), continuous education also surfaced as insufficient at many of the facilities, although not pertinent to all. A pharmacist (P#23) from a district hospital (F15) confirmed: “*Yes, it* [continuous education] *definitely is lacking at F15*.” The importance of continuous education was reverberated in the comment received from P#63, a pharmacist from a national central hospital (F23): “*Because again the limit* [referring to making a recommendation pertaining to a patient’s treatment] *is that a lot of us aren’t as fresh out of varsity so it’s very difficult to go back ten years and implement what you learnt then with now because things obviously have changed*” Another comment received from a specialist (P#26; F15; district hospital), reiterated the importance of continuous education: “*I think in my department a lot of the MOs* [medical officers] *are older and possibly haven’t updated their knowledge. That’s all I can put it* [referring to inappropriate prescribing] *down to.*” A specialist shared that “*where I did my undergraduate training it was phenomenal, it was really, really good. I’ve had very little training since, to be honest*” (P#26; F15; district hospital). Continuous education furthermore seems to be neglected for the bulk of prescribers in the PHC environment, as a family physician from a 24-h CHC (P#28; F3), mentioned, “*…but them* [nurses] *being not doctors, they are not doctors—because you have to register to attend, but it’s open to everyone but there’s limited space, so it’s mostly doctors who attend and nurses are left out…*”.

As mentioned, the lack of in-service training does not apply to all the facilities. At a provincial tertiary hospital, continuous education does happen, although it is ward specific. This speaks of HCPs taking responsibility to educate fellow HCPs, as illustrated by a specialist who involves all the staff in a particular ward:

*“…because we give education not just with dosages etc., but infection control that everyone involved in the care of a patient needs to know—small things like the catheter mustn’t be lying on the floor or on the bed. We also reiterate these points, for example, to sisters before giving drugs, examine the drip and see if there’s any inflammation or erythema or anything going on there, or alert and teaching them to prompt the doctor to look for those things if they’ve noticed it. So, not just in a formal sense of how, even how the antibiotic chart works but the small everyday things that continuous education, if it’s really helpful, makes a difference. And from the physiotherapist to the sister to the OT, everyone who comes to see the patient, remind them to be aware of that, and they do alert us to problems that we sometimes have missed, so that everyone involved in the care of a patient having that basic training really makes a difference”*.

(P#64; F19)

#### 2.3.3. Communication and Feedback

Effective communication and feedback aids to continuous education. The results of Phase 1 reflected 65.4% *(n =* 17*)* of HCPs receiving feedback and while exploring this further during Phase 2, it emerged that the value of communication and feedback are necessary and appreciated.

*“The pharmacy side does come and sometimes they revise, and they want to know the reasoning behind certain prescriptions, the duration of the dosage and I think that forms a kind of feedback function because a lot of times we may learn from that, that we should adjust our prescribing behaviour at times”*.

(P#27; community service MO; F3; 24-h CHC)

*“… there has to be more active communication in terms of educating everyone that this is what our goal is of our hospital, what our aim is and having access to all the information so that everybody’s on the same page”*.

(P#23; pharmacist; F15; district hospital)

The method of communication and providing feedback might not always be as effective and remains a challenge, as a pharmacy manager at one of the national central hospitals explained, that “… *you do training, you send e-mails, you almost repeat yourself constantly. But it’s very difficult to manage again, the human factor. I don’t think sometimes people even read their e-mails, although it’s been highlighted that it’s very important*” (P#9; F6).

The shortage of manpower definitely has a negative impact on effective communication and feedback as reverberated by an infectious diseases specialist (IDS) (P#8) at a national central hospital (F6), mentioning *“… we used to be on a monthly basis, but now unfortunately it’s only on a quarterly basis* [referring to AMS meetings] *and that again is because of limited human resource…we have a program and I think there’s significant problems with it”.*

#### 2.3.4. Public Awareness

The need for public education was widely acknowledged, but barriers e.g., manpower, remains a challenge. The operational manager (P#53) of an 8-h CHC (F22) mentioned they “*have so many other campaigns that we always have to be on top—handwashing, HIV, M&E* [monitoring and evaluation]*, IPC* [infection prevention and control].” The pharmacy manager (P#5) of a 24-h CHC (F26) mentioned human resources as a factor for non-compliance: “*I don’t think at a CHC level they will have the extra staff or the means to do that, but they need it, we need it* [public health campaigns]”. Additionally, at district level, a specialist agreed that “*…definitely, public health education is important. I don’t know how we get the messages out into the community, but it definitely needs to be done”* (P#25; F15).

### 2.4. Involvement of Management

During Phase 1, the majority (57.7%; *n* = 15) of participants were in a managerial position of whom 80.8% (*n* = 21) were supportive of AMS and 65.4% (*n* = 17) involved in ASP activities (33). During the sequential Phase 2 however, only 22.9% (*n* = 19) of FGD participants indicated that they had managerial responsibilities and the lack of overall management involvement surfaced as a considerable problem. This does not only apply to the visibility of management, but “*people* [managers] *not being available. So, that’s also a real concern. But I don’t see decision-making coming from our leaders within the institution. I’m very sorry to say that*” (P#9; pharmacist; F6; national central hospital). Management are not that supportive of AMS and might not understand the severity of AMR as “*there are other areas that take priority, like your OPD…management will be worried about the waiting times—patients waiting for a very long time—and then there are complaints about quality and then everyone is moved from where they are into OPD, instead of concentrating on other areas*” (P#2; pharmacist; F7; national central hospital). The pharmacy manager at a national central hospital said “*You’ve got committed and dedicated people within the institution. Unfortunately, they can’t be the only gatekeepers within the institution. It’s a whole team effort, and it starts from top management; that should be filtered down to all the lower category workers as well*” (P#9; F6). According to a specialist (P#11) at a national central hospital (F6) “*management is definitely not serious with the infection control at this institute because they’re not visible at all. And even if they’re not visible you don’t see any infection control practices being implemented despite all the complaints and the mortality and morbidity issues that are raised because of infection control issues*”.

Pertaining to the matter of IPC, some facilities are in desperate need, as explained by one of the pharmacy managers (P#5) at a 24-h CHC (F26): “*…it is not always active that committee…so I think because the high turnover in staff, what happens is then whoever is on the committee and then they are not there anymore and it falls apart…*” Similarly, an IDS (P#8) at a national central hospital (F6) said, “*Our IPC is lacking. Their* [IPC representatives] *presence is lacking on all of our committees and on all of our stewardship ward rounds.*” At one of the provincial tertiary hospitals (F19) the quality assurance coordinator (P#77) mentioned that, “*We don’t have a qualified infection control person in the hospital currently, so it’s functioning under the quality assurance*”. 

Management involvement is imperative, illustrated by the words of a clinical pharmacist, *“…you must have people who are supporting you. I could do nothing in this hospital if I don’t have the whole support system standing with me and our clinical manager that is supporting me all the way”* (P#62; F5; district hospital). The support of management can clearly make a definite difference, which is appreciated by the HCPs who must perform their daily duties: *“I think we are actually a bit fortunate in this hospital compared to other hospitals that we have support from the pharmacy inside here, support from the clinicians to actually try and make changes to implement the prescription chart. Whereas speaking to colleagues in other hospitals, they don’t have that support, actually in some cases it is non-existent”* (P#3; pharmacist; F7, national central hospital). Such support from management, together with dedicated HCPs has led this facility to be the most compliant among the eight national central hospitals that participated in Phase 1 [48.5%–90.9%] [33].

### 2.5. Antimicrobial Champion

Upon asking what the role of an antimicrobial champion entails, a pharmacist at a 24-h CHC explained *“… and then being a champion, promoting the program, getting everybody involved, and up to speed with the latest recommendations, and I suppose adherence to the guidelines too. The guidelines, correct antibiotics for the correct indication”* (P#30; F3).

The desire exists to have someone taking responsibility and be accountable for AMS and ASPs, which has been identified as being deficient in the current situation. The comment made by P#74 (specialist; F19; provincial tertiary hospital) echoes this:

*“I think the most important gap in all of this antibiotic stewardship is not having currently at least a weekly ward round with an infectious diseases specialist or someone who is orientated or coming from that background* [e.g., an antimicrobial champion] *… because they will question all the decision-making and educate on a weekly basis not only to consult on difficult patients where there needs to be a more specialist opinion, but they’ll look after the small things and they will orientate everyone. They’ll get everyone on board.”*

By having a dedicated antimicrobial champion will perhaps ease the workload burden. An IDS at a national central hospital (P#4; F7) said “…*here is not a huge number of people available to do these ward rounds and the problem is that everyone has duties within their own departments as well. It’s not that the AMS committee is separate and that’s their only thing that they’re doing”.*

### 2.6. Multidisciplinary Collaboration

The importance of interaction among various disciplines was consistently acknowledged throughout the discussions. During Phase 1, 84.6% (*n* = 22) of participating facilities stated that different disciplines do work together to reduce AMR. In facilities where multidisciplinary collaboration and support were evident, the compliance with the Framework was also better, e.g., a district hospital (F5) was 70.6% compliant and the clinical pharmacist (P#62) confirmed “*getting very good support from our clinical manager*, [person’s name], *who is currently acting CEO, as well as the nursing staff and the doctors. So, if I realize there’s something wrong I will contact them, but they also contact me if they are not sure about the dosage or anything, they communicate with me*.” This also applied to another district hospital (F15) “*Because Dr X sits with us* [pharmacists] *after we’ve done the guidelines to go through the guidelines to see if there are things that we need to change, and even Dr Z, who is the microbiologist*” (P#24; pharmacist). This facility was 88.2% compliant with the Framework [33].

### 2.7. Collaboration with Microbiology

From the FGDs, it became evident that interaction with microbiology mainly happens at higher level public health care facilities. A microbiologist (P#14) confirmed “*whether it’s in medical, general ward whatever, we phone out* [inform them of] *the positive culture results for the blood cultures*” (F6; national central hospital). Another national central hospital (F7) highlighted the value of a functioning microbiology lab, saying they “*receive every single ward on Friday and we get a report of what resistant organism had been cultured—ward per ward….and we are the only hospital in the country that gets ward by ward weekly reports as to what resistant organisms we are seeing*” (P#1; critical care specialist). Unfortunately, effective interaction among microbiology and CHCs are very little. P#18 stated they “*… don’t have the privilege of seeing how bad we’re doing in terms of resistance. So, I don’t know, you mentioned something about an antibiogram. If we maybe had that available to the whole clinic, the whole team of health care workers, we will know how we’re doing …*” (P#18; job description undisclosed; F14; 8-h CHC).

### 2.8. Antimicrobial Stewardship Activities

Antimicrobial stewardship has evolved over the past couple of years. A pharmacist (P#24) who has been working between 5-10 years at the particular district hospital (F15) explained that being involved with AMS, there has been progression over the last two years, and although it is still evolving, shortcomings have been identified: “*… like we spoke about the antimicrobials, the facility reports, the antibiograms. I mean, that’s a process that has just started, I think last year. And it is very generalized. But it is something where we’ve seen changes in the patterns already with the few presentations that we have had. But that’s something that needs to be more streamlined.*” 

Facilities could also see the actual difference and impact AMS has brought to their facilities:

*“… and we started with the antibiotic stewardship, the first month or two we were on 10% to 30% and now some months we get 80%”*.

[referring to correct antimicrobial prescribing] (P#52; operational manager; F22; 8-h CHC)

AMS has however not yet been implemented at each and every facility as stated by P#39 *“We haven’t had such initiatives* [referring to AMS and ASP] *over the period that I’ve been here—for 11 years. We haven’t seen initiatives of that nature of any kind”* (registered nurse; F4; 24-h CHC).

#### 2.8.1. Policies, Guidelines and the Revision Thereof

Albeit that only 57.7% (*n* = 15) facilities participating in Phase 1 had guidelines regarding antibiotic choices in place, it is still met with resistance:

*“Some doctors are resistant and there are some that are willing to accommodate and say okay we’ll change it to correspond to the guidelines. Because we have disseminated our guidelines throughout the hospital in electronic format, so there is access to it in all the wards”*.

(P#24; pharmacist; F15; district hospital)

Some facilities had specific policies in place such as different antimicrobials having a level as to which healthcare professional is allowed to prescribe it. P#24 (pharmacist; F15; district hospital) explained “*for carbapenems, they require a consultant to sign the antibiotic prescription. So, they have been reviewed by a consultant.*” This measure might become defeated as experienced at a national central hospital (F7) when “*they* [referring to consultants] *send their junior interns to come and get the permission.*” These policies present with further challenges e.g., prescriber autonomy as explained by a pharmacist (P#3) from the same facility: “*You’ve got, each consultant has their own way of prescribing antibiotics and they feel like if a certain one has to authorize it, they feel like, they’ve intruded on their territory and why should I not be able to authorize, whereas my colleague who’s maybe on the same level as me, or slightly senior, and then it creates also issues with that*.” On the other hand again, not everyone has the same knowledge of, and awareness about, the guidelines:

*“I think in most instances it’s a lot of interns prescribing. They are not aware of the guidelines maybe, so they won’t even look at the guidelines”*.

(P#23; pharmacist; F15; district hospital)

*“If someone is coming from outside, like if it’s a locum nurse or doctor, then they often, don’t know what the latest guidelines are, or... they don’t have the mobile app *[with the guidelines]*... they haven’t worked in primary healthcare for a long time”*.

(P#52; family physician; F22; 8-h CHC)

The pharmacist plays an important role by ensuring HCPs prescribe according to guidelines because *they “have to check before we dispense the script if it’s in accordance with the guidelines. That’s when we also can intervene and communicate with the doctors regarding the prescription … as to why they’re using that and what is in our guidelines to guide them as well”* (P#24; pharmacist; F15; district hospital).

Regular revision of guidelines as a basic AMS principle was done in less than half (42.3%; *n* = 11) of the facilities during Phase 1. Although revision of guidelines is a requirement for effective AMS, concerns have been raised regarding the actual overseeing of the process:

*“… we have lots of problems with nosocomial sepsis and we’ve had a look from 2013 at all our patients who have been admitted there and seen what has been cultured from various samples and correlate it with the clinical picture of the patient and what antibiotic was sensitive to and what was the outcome of the patient. We’ve made up our own guidelines on the antibiotics that we used based on that. But it’s not ‘policed’ by a suitably qualified person or reviewed or compiled. It’s our own solution to the problem that we face*”

(P#74; specialist; F19; provincial tertiary hospital)

#### 2.8.2. Antimicrobial Prescription Charts

Specific interventions to improve antimicrobial use include the development and use of an antimicrobial prescription chart. The value of using the prescription chart is recognised, e.g., “*having people interested in antibiotic stewardship and getting them geared and the nursing staff, and all of us on the same page…*” (P#24; pharmacist; F15; district hospital). The antibiotic prescription chart is also used as a tool to do auditing: “*We do audits daily—the pharmacist interns do audits daily in the wards. We’ve developed an antimicrobial prescription chart that’s unique to F15*” (P#24; pharmacist). A similar idea has been implemented at F14 (8-h CHC): “*Our IPC manager has created a data collection tool that we use. For example, whenever we prescribe antibiotics, we will write in the diagnosis and the antibiotic. That is the analysis that we do to see which antibiotics we prescribe and what it may be prescribed for*” (P#15; infection control coordinator). These types of interventions clearly function to gate keep appropriate antimicrobial prescribing and definitely enhances AMS, as alluded by a critical care specialist (P#1): “*It is not just a matter of filling in the form. When we* [signatories] *see those forms they usually are wrong doses, wrong antibiotic for that situation, inappropriate antibiotic, that they don’t actually need an antibiotic.*”

Although this antibiotic prescription chart is a valuable tool, it has its own challenges as “*it is a bit difficult in the wards because they don’t always fill in the information …*” (P#66; pharmacist; F23; national central hospital). Incomplete antibiotic prescription charts manifest in many of the facilities and cannot be utilized to its full potential because “*… say for instance due to the incompleteness of the antibiotic prescription chart, you cannot get that information... and the pharmacy also don’t have time to phone the doctor*” (P#9; pharmacy manager; F6; national central hospital). P#8 (IDS; F7; national central hospital) agreed that such a chart could be of immense help in terms of ASP “*but there’s major issues with that, because … we’ve got shortage of resources issues in the pharmacy, technical resources.*” A critical care specialist (P#1) from the same facility echoed “*… now that is still a problem and they are still not using them* [antibiotic prescription charts] *as well as they ought to and certain people are signing that probably should not be signing as well in terms of those charts, and that still requires ongoing education with regards to the use of antibiotics.*” It is however good to see the initiatives taken to overcome some of these problems:

*“I also developed in the beginning a form with different colours that say to the doctors and the nurses who must do what, what information must be where and everything”*.

(P#62; clinical pharmacist; F5; district hospital)

#### 2.8.3. Confirming Indication

A mere 50% (*n* = 13) of participants in Phase 1 said that they confirm the indication against each antibiotic prescribed. This is an important process measure as prescribers omit to provide this information in many instances. A pharmacist (P#12) at a national district hospital (F6) confirmed that “*the majority of our antibiotic prescriptions don’t actually have an indication or a diagnosis …and some of our antibiotics are level 4. You have to have a micro report, so we have to phone.*” The HCPs participating in the FGDs mostly followed up in the event of an indication omitted, as mentioned by one of the pharmacy managers at a 24-h CHC (P#5; F26;): “*… the diagnosis should be there, very clearly written so that is why we will often liaise with the doctor.*” 

#### 2.8.4. Requesting of Blood Cultures

Another important aspect of AMS is to request blood cultures before initiation of an antimicrobial and collaboration with microbiology. During Phase 1, even though 63.6% (*n* = 7) of facilities had the request for culture and sensitivity testing established, it was obviously lacking at many facilities, especially at PHC entry level. A clinical pharmacist (P#62) at a district hospital (F5) mentioned the budget constraints: “*The culture is not sent because it’s too expensive. We also just have a limited budget*”. A functioning microbiology laboratory was only available at 53.8% (*n* = 14) of the facilities, and without the support of this important discipline, key process measures cannot be carried out:

*“When we do our audits, you’ll see that most of them don’t do a culture, because we have a box in our antibiotic *[prescription chart]*—‘Have you sent for a culture before initiation or not’, and most have ticked ‘No’. So, they’re just treating empirically”*.

(P#24; pharmacist; F15; district hospital)

The following comment reverberates the lack of collaboration with microbiology as mentioned earlier on and the omission of requesting cultures:

*“… instances where you look back in a patient’s history and you see that they’ve been taking one antibiotic for a number of weeks or months with no change for the same condition and then that warrants another review from the clinician”*.

(P#17; pharmacy manager; F14; 8-h CHC)

#### 2.8.5. De-Escalation

*“If a patient has been on the medication for like two days, then most of us have access to NHLS* [National Health Laboratory Services] *and we will then look at when we can de-escalate”* (P#63; pharmacist; F23; national central hospital). This is good practice, but due to ‘no requesting of blood cultures’, as alluded to above, the assumption is that de-escalation does not apply at many of the public healthcare facilities.

#### 2.8.6. Audit and Feedback

The results of Phase 1 reflected that a mere 38.5% (*n* = 10) of facilities undertook audits on antimicrobial use and provided feedback. It is evident that regular auditing of antimicrobial use made a positive impact at a district hospital (F5): “*I’m checking whether the doctor prescribed the correct medication for the correct diagnosis and the correct dose and everything. The second one is, did the patient receive all the antibiotic doses as prescribed and then were there any fields missed on the prescriptions. I started a year ago with that. Our percentage compliance at that stage, was 41%. Our compliance is now 93% for the previous month*” (P#62; clinical pharmacist). At a district hospital (F15) the pharmacist (P#23) explained their prescription auditing and feedback process as part of antimicrobial stewardship *‘… where the charts are being audited and where they’ve* [doctors] *been questioned about the indication for prescribing. I think that’s making them realise—because the fact that we’ve even changed the antimicrobial prescription charts, alerts to them that this is something of great importance and they should be very aware of it*” 

At one of the 8-h CHCs (F14), a data collection tool was developed to audit prescribed antimicrobials vs. indication, however, not everyone is acquiescent which impacts negatively on feedback. The CEO (P#16) explained it as follows: “*It’s just that in other departments, like the primary healthcare department or our HIV department, the clinicians are not so compliant because of the multitude of administration work that they have to do, so it often gets left behind. Even though it’s a user-friendly tool—it’s just a ticking tool—so you just tick the diagnosis and you tick the antibiotic that you’ve used.*”

#### 2.8.7. Cumulative Antimicrobial Susceptibility Report (Antibiogram)

Only 38.5% (*n* = 10) of the facilities have produced a cumulative antimicrobial susceptibility report in the previous year at the time of taking part in Phase 1. Although a microbiologist (P#81) from national central hospital (F7) said that they provide these reports “*to ICU and H/C* [high care] *areas where we do rounds*”, it does not seem that easy at CHC-level where a pharmacy manager (P#5) explained: “*I tried doing a … get a report from the labs with regards to us and our profile because, I was there last year* [referring to an AMR workshop] *and they alerted me to that we can actually get that. They* [microbiology] *were not happy to do it here, but they did it for me once off*” (F26; 24-h CHC). A specialist (P#74) from a provincial tertiary hospital (F19) said that “… *I haven’t encountered a compiled antibiogram that’s done for the whole hospital from a set of cultures from a certain ward done by microbiology or by the lab*”. The pharmacy manager (P#17) at an 8-h CHC (F14) realizes the importance of this process measure and confirmed “… *at this stage we are more in laying down the foundations for us to get our systems in place in such a way that we do get the antibiogram*”.

#### 2.8.8. Monitoring of Antimicrobial Consumption

Regarding antimicrobial consumption monitoring, Phase 1 results showed that only 38.5% (*n* = 10) of facilities conducted audits on antimicrobial consumption. It appeared as if this important process measure was easier accomplished at higher levels of public healthcare facilities. The pharmacist (P#63) at a national central hospital (F23) confirmed that “… *it is at the management level. So, they keep an eye on what gets used all the time because of money*”. Another pharmacist (P#71) from a provincial tertiary hospital (F19) said that “*as soon as we see an increase in consumption … we do investigate to see where the problem is … is there an increase in patients, were there more cases… and we do ask for the lab’s feedback as well*”. However, the pharmacy manager (P#5) at a 24-h CHC (F26), highlighted their challenges and said “*it is very difficult in the province… logistical issues … infrastructure issues, we do not have JAG* [an electronic system] *here so it is very difficult for me to get that numbers and to get a DDD calculation so that I can see, you know exactly compared to other facilities where do we stand*”.

To monitor the consumption of antimicrobial use is further a challenge when records are not kept. The CEO (P#16) of an 8-h CHC (F14) confirmed, “*... we don’t have enough space for filing. If we open files for all of the patients, within a month we are going to run out of space”*. Another 8-h CHC (F14) had a similar challenge, as the nursing manager (P#13) confirmed, “… w*e don’t keep records. If a patient came a month ago and collected Amoxil^®^* [amoxicillin] *and they come with the infection again, tonsillitis or whatever, we don’t have records of the previous visits to say, because they make new cards at each visit, so we don’t have a card to say—she got Amoxil^®^ now she’s coming again with the same infection”.*

It was evident that insufficient infrastructure, restricts effective AMS and ASPs:

*“At times our files, sometimes previous clinical notes are lost or misplaced so we are not able to follow up the patient’s history chronologically. So, at times something might have been prescribed in the past and it wasn’t noted down, because the patient has had a replacement file, so then it might be prescribed again, because our patients are also unaware sometimes of what medication they’re taking”*.

(P#27; community service MO; F3; 24-h CHC)

*“Many times I’ve seen with the filing office a patient will be prescribed a medication, the history is lost and they give the patient a new file. How can this patient be properly followed up?”*.

(P#44; pharmacist; F4; 24-h CHC)

An IPC nurse (P#76) from a provincial tertiary hospital (F19) once again highlighted manpower as a hindrance *“But also then again, it’s very important for people to fill those positions that is very critical… currently that we cannot do without. Even the surveillance would be up to a better standard so one can work more to a point of prevention—better than curing”*.

## 3. Discussion

We believe this study appreciably extends our understanding of the current situation in South Africa’s public healthcare sector regarding the Framework to help reduce AMR rates. This is important especially in sub-Saharan Africa given the multiple ongoing activities introduced in South Africa to improve AMR including the initial guidelines to implement the Framework which were made available during June 2017 [23,48,66,67]. Phase 1 of the study reflected an overall compliance with the Framework of 59%, ranging from 12.9% (CHC-level) to 90.9% (national central hospital). The average compliance among CHCs was 37.9%, while at referral and national central hospitals, 66.8% and 73.5% respectively [23,33]. It was evident that entry level public healthcare facilities are hindered by many challenges to implement the Framework effectively. This is not surprising in view of their resource constraints. Whilst higher level facilities are not without challenges, there appear to be fewer obstacles to overcome when compared to entry level facilities. Consequently, the discussion will focus on the broad strategic enablers and objectives of the Framework while highlighting other challenges impacting on its ultimate goal. As such, offer guidance to all key stakeholder groups in South Africa and wider as they seek to implement agreed activities as part of their country’s NAPs for AMR.

Apart from legislative and policy reform, education and communication form part of the strategic enablers and the foundation of the Framework [68]. Both the National Drug Policy and the Health Professions Act (Act 56 of 1974) reinforce education and training as there are concerns that insufficient knowledge regarding the rational prescribing of antimicrobials will compromise the value of such initiatives [68,69]. This is important as we are aware in some LMICs that a key source of information and influence regarding antimicrobials is from pharmaceutical companies, which this includes sub-Saharan African countries [49,70,71,72]. It was evident that this very important enabler was lacking at most of the facilities, especially at the entry level. Clinical nurse practitioners are often ignored in campaigns, or continuous professional education activities, to increase awareness about inappropriate antibiotic use and the ever-increasing AMR [38,73]. This is a concern as typically they are the principal prescribers in PHCs in LMICs [38,41]. It is concerning that most of the targeted facilities were not providing continuous training on antimicrobials and AMR, and when it does materialise, training is usually at facilities providing higher level of care services and very much dependent on dedicated HCPs taking the lead. This confirmed the low percentage (42%) of HCPs who indicated during Phase 1 that they receive continuous education on local AMR [33].

The guidelines for the containment of AMR in South African hospitals aim to address AMS components with in-service training and behavioural change as its foundation [74]. Apart from limited human resources and lack of expertise, both playing a definite role, it appeared however that in-service training is offered selectively for certain disciplines in many of the facilities. Specialised disciplines such as Medicine and Paediatrics, who are managing infectious diseases on a daily basis, are mostly targeted for training, although, because of their expertise they are most likely to prescribe antimicrobials appropriately. However, this is not always the case in other sub-Saharan African countries or when reviewing compliance to guidelines to prevent surgical-site infections in pediatric patients and adults undergoing surgery in South Africa and wider [39,56,75,76]. Consequently, other disciplines should also be targeted for in-service training, or prioritised for training, especially where the availability of resources is a challenge. The current focus of in-service training should be addressed and aligned to the guidelines for the containment of AMR in South Africa and wider. This includes all general clinicians and nurses who are involved in the day-to-day care of patients with infectious diseases across sectors [21,35,74,77]. This is especially important in sub-Saharan Africa given their high prevalence rates of infectious diseases and variable knowledge of AMR and ASPs [48,50,67,78].

A great concern in our study is that educating the public about antibiotics and AMR is scarce and performed haphazardly during consultation. This may be due to time constraints as seen in PHCs across LMICs [35,41], and may help explain why only 11.5% of facilities in our study initiated public health campaigns during Phase 1. An acute need exists to create awareness among the general public regarding antibiotic misuse in South Africa and wider especially for predominantly viral infections such as upper respiratory tract infections [21,35,73]. Public health campaigns should be designed to raise public consciousness about important health issues such as AMR, and targeted audiences should be educated regarding imminent health threats that might be harmful to them. However, we are aware that there are currently limited publications on the cost-effectiveness of such strategies in LMICs versus high-income countries, which needs to be addressed going forward [21,35].

Public healthcare facilities destitute of resources remain an ongoing challenge to the provision of an agreed level of healthcare. Apart from budget constraints, lack of human resources was a common phenomenon in all our targeted facilities, with a heavy workload creating a viscous circle of more work overload. This is similar to other LMICs [21,47]. The limitations of infrastructure stretch far and wide, from too little space to keep patient files, affecting effective AMS, to too small or no space to do continuous education, to no handwashing soap and emollients, which also impedes IPC in general, subsequently having a negative impact on AMS.

With limited microbiology results available, proper monitoring of antimicrobial consumption and surveillance data and performing AMS, becomes a problem [79]. However, we are aware this is changing in a number of LMICs including sub-Saharan African countries as NAPs progress with the urgent need to reduce high levels of AMR as well as greater investment in healthcare [19,21,56,80]. Participants in our study reiterated that the absence of an information feedback system with the availability and integration of microbiology laboratory results at prescriber and dispensing level had negative implications for the practical application of AMS. Having said this, the majority of infections seen in ambulatory care will be viral in origin; consequently, this should be borne in mind during consultations [21,35]. This is especially important in the current COVID-19 pandemic where only a limited number of patients with COVID-19 in ambulatory care have concomitant bacterial or viral infections [21,81,82].

Insufficient IT, particularly access to Wi-Fi, free data and technical support, is another considerable resource issue across LMICs including sub-Saharan Africa. Whilst guidelines are available through a mobile application in South Africa, they cannot be accessed by the vast majority of prescribers in PHC facilities due to the unavailability of free Wi-Fi or data. As a result, and in the absence of Wi-Fi, prescribers refer to outdated guidelines which are still available in hard copy at these facilities. All of these challenges impact negatively on the final goal of the Framework, which is to improve patient outcomes. Within modern healthcare systems, IT is extremely important for the effective implementation of ASPs including providing up-to-date and easily accessed guidelines to improve the management of infectious diseases [37,83]. The success of many AMS tools and interventions rely on the availability of IT such as clinical decision support systems, smartphone applications and electronic health records [83], and we will be following up on this is the future.

The findings highlighted that the involvement and support from hospital management is of utmost importance for the success of ASPs. This is a concern as some facilities surveyed experienced major challenges in terms of lack of management involvement, visibility and support. Facilities supported by management were clearly more compliant with the Framework as per Phase 1 [33]. Strategies should be developed to engage and establish ongoing relationships with hospital and PHC management and health system administration to support AMS initiatives and ensure allocation of the necessary resources to fully undertake ASPs in South Africa and wider [84]. This is starting to happen across Africa but from a low base [80].

It was particularly concerning that ASPs and AMS activities are not yet implemented in all the surveyed facilities in South Africa following the national directive, and then mainly at CHC-level. This has important implications not only for South Africa but wider across sub-Saharan African where a number of initiatives are inly just being introduced within NAPs. Encouragingly, the majority of facilities surveyed in South Africa did have a functioning pharmaceutical and therapeutics committee (PTC) in place and with AMS as well as generally enhancing the rational use of medicines through assessing prescribing against national guidance, a key function of the PTCs [85,86,87]. Some actions are performed to support optimal antimicrobial use such as having policies and guidelines in place with regular revision occurring [21,37]. However, concerns exist if current guidelines are not regularly reviewed or compiled by suitable qualified personnel [37,88,89,90]. This again ties up with concerns with the lack of human resources and scarcity of expertise among surveyed facilities. Applying guidelines is an intervention to support clinical-decision making and enhance care [90,91,92,93]. It is however important to include the opinion of key personnel from medical specialties when developing policies and guidelines for prescribing, including antimicrobial prescribing, to enhance guideline acceptability and use [37,89,94,95]. Compliance to current guidelines remains a limiting factor among all surveyed facilities in South Africa, which needs to be addressed going forward as we have seen higher adherence rates to antimicrobial guidelines in other facilities in sub-Saharan Africa [67,93]. An important consideration for future interventions is that the success of AMS is reliant on changes in the behaviour of individual prescribers. Consequently, a key facet of ASPs would be to target improved and sustained individual prescribing behaviour [96].

In the healthcare facility, IPC is a core component of overall quality control and the mainstay of containing AMR [20,97]. Whilst 84.6% of facilities confirmed a functioning IPC during Phase 1, it became very apparent that serious challenges exist at many of the facilities in South Africa. Once again, these challenges relate to lack of management involvement, deficient human resources, inadequate set-up, and shortage of microbiology support, among others, similar to other LMICs including sub-Saharan African countries [47].

We are aware of a number of limitations with this study. Despite a relatively good response rate for the survey conducted in Phase 1, not all provinces participated in Phase 2 and facilities were not representative of the entire country and healthcare system. However, we believe the mixed-methods approach with FGDs in Phase 2 enhanced the robustness of the data collected and gave multiple opportunities to explore the quantitative data collected in Phase 1. Consequently, we consider our findings robust enough to provide guidance and direction to develop future interventions to address the challenges identified in South Africa and potentially wider, thereby strengthening ASPs and prevent further development of AMR.

## 4. Materials and Methods

### 4.1. Design

A 2-phased mixed methods study with a sequential explanatory research design was employed. Phase 1 used a quantitative descriptive design, with a self-administered questionnaire, to assess compliance with the AMR National Strategy Framework among public healthcare facilities in South Africa [33]. Phase 2 employed a qualitative methodology with FGDs and in-depth interviews, aimed at exploring the results obtained during the first phase. Furthermore, the results of Phase 1 and the findings of Phase 2 were combined (mixed) for overall understanding and interpretation. Subsequently, recommendations were formulated for the development of targeted interventions to enhance the role, impact and activities of ASPs and thereby the sustainability of the Framework. We believe employing both research approaches enhanced the integrity of the findings and therefore the credibility of recommendations to provide future direction [98].

### 4.2. Setting

Since the majority of South African households make use of public sector facilities as a first option to access healthcare [30], this study was conducted among public sector facilities. Study sites included all 9 national central hospitals in South Africa, 10 referral hospitals (at least one per province) and 18 CHCs (2 per province). The percentage compliance with the Framework for each participating facility was calculated based on the results of Phase 1 [33], and subsequently used to identify facilities for the sequential qualitative phase (Phase 2). 

### 4.3. Participants

**Figure 2:**
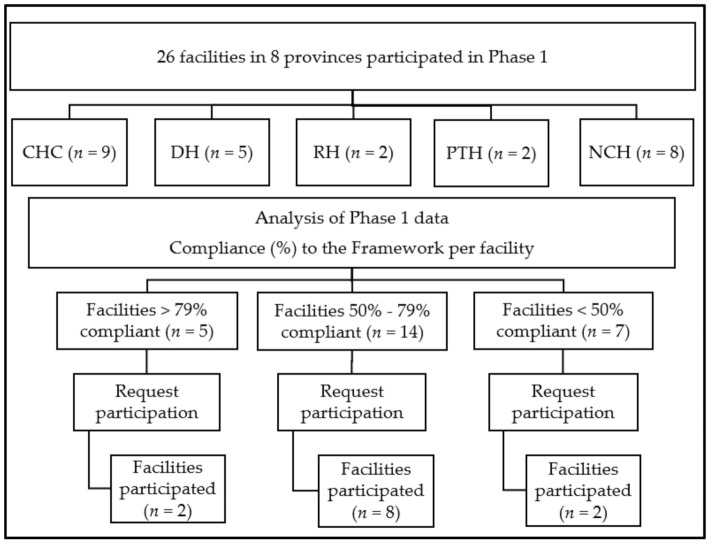
Study sites for Phase 1 [33] and Phase 2. CHC = Community Health Centre; DH = District Hospital; RH = Regional Hospital; PTH = Provincial Tertiary Hospital; NCH = National Central Hospital.

The target study population for Phase 1 comprised one invited healthcare professional per facility, who was permanently employed and knowledgeable on and/or actively involved in AMS activities. Facilities that participated in Phase 1 were categorised according to their percentage compliance with the Framework [33], and invited for participation in Phase 2, ensuring balanced representation of facilities from each compliance category. Participants for Phase 2 (qualitative) were recruited purposively [99], to ensure information rich participants with maximum variation. Eight facilities with 50%–79% compliance to the Framework participated, and two facilities each from the category above 79% compliance and below 50% compliance (Figure 2).

For the purpose of Phase 2, the HCP who completed the questionnaire during Phase 1 [33] was requested to recruit 6–8 participants from his/her facility to participate in a FGD, based on their knowledge of AMR and involvement in ASPs. To ensure representation from all aspects of AMS, inclusion of participants from various disciplines, including members of the PTC and ASP, IDSs, microbiologists, practitioners, pharmacists and clinical nurse practitioners (CNPs) were requested. Where a FGD was not possible at a particular facility, an in-depth interview with a key person in the field of AMS was requested.

### 4.4. Data Collection Instruments and Process

Detail on the data collection for Phase 1 has been described previously [33]. In summary, a web-based application, adapted from a point prevalence study [100,101], enabled participants to complete an English questionnaire via any mobile or desktop device with internet connectivity. The Framework guidelines served as the basis for the questionnaire, which related to three critical governance structures: IPC, PTC and AMS, with AMS being the focus of this paper.

In Phase 2, various strategies were used throughout data collection and data analysis, to ensure the trustworthiness of the findings [102,103]. A focus group- and in-depth interview guide with open-ended questions to facilitate discussion, was developed for the different categories of healthcare facilities. The guide was adapted for each particular facility according to their results obtained during Phase 1 [33]. FGDs and in-depth interviews were conducted in an available venue where privacy was ensured, and facilitated in English by DE. During the introduction, an overview of the study and the purpose of the discussion were provided, and reiterated that the aim of the discussion was not to reach consensus. Each discussion was recorded using a digital voice recorder to ensure dependability and transferability with a track record of the data collection process. To maintain confidentiality each participant was allocated a unique identification code, which was used during the discussion as identification, instead of using real names. This ensured anonymity during transcription, analysis and reporting. Participants signed the attendance register, which was also utilized to collect participants’ basic demographic data in terms of job description and duration in current position. Disturbances and interruptions were kept to a minimum as far as possible.

Where in-depth interviews were conducted, the same principles were followed. During the FGDs introductory and basic questions included in the interview guide were adapted and elaborated on, pinpointing data relevant to the study [104]. The credibility of the findings was ensured through spending sufficient time with FGD participants to ensure rich discussions and to gather as much data as possible, whilst taking cognizance of the time, as participants are likely to suffer from fatigue when discussions are lengthy [105]. Whenever unclear responses had to be verified or when further explanation was required, appropriate probes were used which enabled an in-depth understanding of ASPs, the successes, challenges, and possible shortcomings thereof. This in turn highlighted any interventions needed for the Framework to be successful in restraining AMR. 

### 4.5. Data Management and Analysis

As described elsewhere, for the purpose of Phase 1 data analysis, the number of questions served as the denominator and the number of positive (“Yes”) answers, indicating compliance with the Framework, served as the numerator [33]. Wherever a question did not apply to a specific facility type, the denominator was adjusted accordingly.

Recorded FGDs and in-depth interviews from Phase 2 were transcribed verbatim by an experienced transcriber and each saved as a Microsoft Word^®^ document. None of the raw data were summarised, rephrased or grammar corrections made. To ensure the credibility and dependability of the data, transcripts were verified for accuracy against the audio-files by the lead author (DE). When required, any uncertain phrases, terms or abbreviations were verified with a particular facility via electronic communication. Each participant was allocated a unique identifier in a sequential manner from one audio-file and transcription to the next (#1–#83). Afterwards, each Microsoft Word^®^ document, including electronic communication, was imported into NVivo12^®^ software, used for qualitative data analysis. 

A step-wise inductive coding and thematic analysis process using NVivo12^®^ was followed [106,107]. Firstly, each transcript was read and re-read multiple times to obtain a clear understanding of the data. DE coded three transcripts, one from each facility type (national central hospital, referral hospital and CHC) through line-by-line open coding, to bring meaning to the text. The second author (JCM) subsequently recoded the data independently, which was followed by discussion, comparison and modification of the codes between the two coders, to develop an initial comprehensive codebook. DE subsequently continued with the coding process for the remaining transcripts. An additional function of NVivo12^®^ software utilized was the capturing of annotations during the coding process, which brought meaning to the text. Codes were organized into categories and sub-categories. Patterns and connections between and/or within categories and sub-categories were identified.

Continuous discussions took place between DE and JCM throughout the data analysis process to ensure consensus about the codes and the development of categories. Categories were subsequently developed into a framework of key themes and sub-themes. Patterns and connections between and/or within themes and sub-themes were identified to provide an accurate reflection of the main concepts, which was based on the responses and perceptions of the participants.

Data transcription and coding took place after data collection in each province. This allowed for identification of any topics or aspects that required further probing or inclusion in subsequent discussions and to determine data saturation. Data saturation was reached after 10 FGDs and two in-depth interviews had been conducted in five provinces since a clear pattern of information emerged and no new information was forthcoming [106]. The use of NVivo12^®^ software furthermore ensured conformability as it provided an electronic audit trail as evidence that interpretations were made based on the data [107,108]. An overall interpretation of themes through a thick, descriptive and narrative approach is presented in the results section.

### 4.6. Ethical Considerations

Ethical clearance for the study was granted by the Sefako Makgatho University Research Ethics Committee (SMUREC/P/316/2017: PG) and permission to conduct the study was obtained from Provincial Departments of Health as well as at facility level. Participation was voluntary and all participants provided informed consent.

## 5. Recommendations

The findings of our study, highlighted a number of important recommendations for South Africa and wider among LMICs. From a national perspective, AMS and ASPs should be reinforced by legislative and policy reform for health systems strengthening. At facility level, although AMS can function either as a subcommittee of the PTC, it can also be a stand-alone committee [84], comprising of dedicated HCPs to manage AMS. As required by the fifth priority area of the National Core Standards (NCS) in South Africa, a qualified HCP should fulfil the role of an antimicrobial champion [23] and needs to be present in each ward [74], which is a necessity and should be extended to entry level public healthcare facilities as well. However, management involvement is imperative also to underpin continuous education, effective communication and public awareness.

Our findings illustrated that to achieve the best patient outcomes, the application of medical knowledge and judgement should be supported by accurate and timely services of a microbiology laboratory. *“Without diagnostics, medicine is blind”* [109]; consequently, the availability and accessibility of microbiology laboratories are critical to enhance AMS activities via improved diagnostics and provision of surveillance data [109,110]. This would subsequently enhance surveillance, support IPC, AMS and eventually prevent AMR.

Future operational projects should focus on the implementation of effective AMS and ASPs at entry level public healthcare facilities in South Africa and wider, especially when keeping UHC and the implementation of NHI in mind. To develop interventions aimed at addressing the challenges identified, thereby strengthening ASPs and restricting AMR, future research should target a number of key areas. These include: (i) Exploring the level of antimicrobial and AMS training at undergraduate level of the various disciplines, with a special focus on the nursing profession as they are the main prescribers at PHC facilities throughout Africa and wider; (ii) Assess continuous education on antimicrobials and AMS in practice and its effectiveness, with a special focus on lower-level PHC facilities; (iii) Determine the impact of a designated antimicrobial champion at ward and facility level as this can render motivation for the need of such a HCP; (iv) Assess the impact of an antibiogram and how guidelines are adjusted accordingly. This can then be related to resistance levels at a particular facility from baseline compared to post-intervention; (v) Determine the value of requesting culture and sensitivity tests from patients returning to a PHC facility with an unresolved illness after receiving treatment previously. This will confirm the need for microbiology support at this level and further confirm AMR at facility level; and (vi) Participatory action research projects aimed at influencing prescribing behaviour.

## 6. Conclusions

Several challenges remain in promoting the appropriate use of antimicrobials among healthcare facilities in South Africa following the implementation of the National Action Plan. Of particular concern is the challenges faced by PHC facilities, being the first point of access to the healthcare system for the vast majority of patients in South Africa and wider. AMS programmes and activities can only be successful once the many challenges faced by public healthcare facilities are addressed. Such challenges are enhanced by the lack of microbiology support, especially at CHC-level, although not exclusively. Enhanced surveillance and diagnostic stewardship are two strategic objectives of the Framework, however, with the limited microbiology support it can be very difficult to accomplish this at entry level healthcare facilities in South Africa. This needs to be addressed going forward in South Africa and wider. Appropriate prescribing and use of antimicrobials at entry level to healthcare services, i.e., *‘getting it right the first time’* is important and a major strategy to combat AMR across LMICs provided the necessary resources and support systems are in place. This includes prescribing guidance where infections are predominantly viral in origin. The use of a newly develop app in South Africa should help in this regard by rapidly reviewing antimicrobial use against current guidance across sectors [64,65].

## Figures and Tables

**Table 1:** Respondents distributed by profession and facility type (*n* = 26) (Phase 1).

Figure		Profession			Total; *n* (%)
		Doctors	Pharmacists	Nursing	
Community health centres		2	3	4	9 (34.6%)
Referral hospitals	District	2	3	-	5 (19.2%)
	Regional	-	1	1	2 (7.7%)
	Provincial tertiary	1	1	-	2 (7.7%)
National central hospitals		-	8	-	8 (30.8%)
Total; *n* (%)		5 (19.2%)	16 (61.5%)	5 (19.2%)	26

**Table 2:** Disciplines represented and duration in current position (*n* = 83).

**Disciplines**	Clinical Associate *	**Medical**	**Microbiology**	**Nursing**	**Pharmacy**	**Expert**	**Undisclosed**	**Total**
Number of participants	2 (2.4%)	18 (21.7%)	4 (4.8%)	20 (24.1%)	28 (33.7%)	9 (10.8%)	2 (2.4%)	83
**Duration in Current Position**								
1–4 years	27 (32.5%)	5–10 years	18 (21.7%)	>10 years	21 (25.3%)	Undisclosed		17 (20.5%)

* Clinical associate = a mid-level worker to assist doctors and the healthcare team mainly in the district/government healthcare system in SA.

## Data Availability

The data supporting the reported results are available from the corresponding author on reasonable request.
